# Costello syndrome model mice with a *Hras*^*G12S/+*^ mutation are susceptible to develop house dust mite-induced atopic dermatitis

**DOI:** 10.1038/s41419-020-02845-8

**Published:** 2020-08-13

**Authors:** Yu Katata, Shin-ichi Inoue, Atsuko Asao, Shuhei Kobayashi, Hitoshi Terui, Aya Inoue-Shibui, Taiki Abe, Tetsuya Niihori, Setsuya Aiba, Naoto Ishii, Shigeo Kure, Yoko Aoki

**Affiliations:** 1 Department of Medical Genetics, Tohoku University Graduate School of Medicine, Sendai, Japan; 2 Department of Pediatrics, Tohoku University Graduate School of Medicine, Sendai, Japan; 3 Department of Microbiology and Immunology, Tohoku University Graduate School of Medicine, Sendai, Japan; 4 Department of Organ Anatomy, Tohoku University Graduate School of Medicine, Sendai, Japan; 5 Department of Dermatology, Tohoku University Graduate School of Medicine, Sendai, Japan

**Keywords:** Interleukins, Atopic dermatitis

## Abstract

Costello syndrome is an autosomal dominant disorder that is caused by germline *HRAS* mutations. Patients with Costello syndrome present craniofacial abnormalities, cardiac defects, and cancer predisposition, as well as skin abnormalities, including papillomas, keratosis pilaris, and eczematous dermatitis. However, the mechanisms underlying the dermatological abnormalities remain unclear. Here, we demonstrated that knock-in mice expressing an *Hras* G12S mutation (*Hras*^*G12S/+*^ mice) are susceptible to develop atopic dermatitis (AD)-like skin lesions, including eczema, pruritus, elevated serum IgE levels, acanthosis, and the infiltration of mast cells, basophils, and type-2 innate lymphoid cells in the dermis, after stimulation with house dust mite allergens (*Dermatophagoides farinae*, Dfb). Reduced skin barrier function, increased proliferation of phosphorylated ERK (p-ERK)-positive epidermal cells, and increased Th2-type cytokines as well as epithelial cell-derived cytokines, including IL-33, were observed in the skin tissue of *Hras*^*G12S/+*^ mice compared with *Hras*^+/+^ mice. Cultured *Hras*^*G12S/+*^ keratinocytes exhibited increased IL-33 expression after Dfb stimulation. PD0325901, an MEK inhibitor, ameliorated AD-like symptoms in *Hras*^*G12S/+*^ mice, showing decreased proliferation of p-ERK-positive epidermal cells and decreased expression of IL-33. Our findings indicate that the epidermis of *Hras*^*G12S/+*^ mice stimulated by Dfb strongly induced IL-33 expression and type-2 innate lymphoid cells, resulting in AD-like skin lesions. These results suggest that the epidermis of *Hras*^*G12S/+*^ mice are prone to development of eczematous dermatitis stimulated with house dust mite allergens.

## Introduction

The skin is a stratified epithelium consisting of several layers of cells in various stages of differentiation. In order to maintain normal skin homeostasis, the proliferation, differentiation, and response of epidermal cells to external stimuli must be tightly regulated^1^. The RAS/MAPK signaling pathway plays a crucial role in cell proliferation, differentiation, and apoptosis^2,3^. A strong activation of the RAS/MAPK pathway in skin is known to result in epithelial cancers and melanoma^4,5^. Pigmented lesions, hyperkeratosis, pruritus, curly hair, and hyperplasia have also been observed in vemurafenib (a BRAF inhibitor)-treated patients^6^. The balance of the RAS/MAPK signaling pathway could be particularly important for epidermal homeostasis.

Noonan syndrome, Costello syndrome, and cardio-facio-cutaneous (CFC) syndrome are phenotypically overlapping genetic disorders, characterized by craniofacial dysmorphia, congenital heart defects, and psychomotor retardation^7^. These syndromes are commonly caused by germline mutations in components of the RAS/MAPK pathway, termed RASopathies, which constitutively activate the RAS/MAPK pathway^8,9^. Of these syndromes, Costello syndrome is characterized by short stature, craniofacial abnormalities, congenital heart diseases, hypertrophic cardiomyopathy, and intellectual disability^10^. Approximately 15% patients with Costello syndrome develop malignant tumors, including rhabdomyosarcoma and bladder carcinoma. In 2005, we identified germline *HRAS* mutations in patients with Costello syndrome^11^. A nucleotide change that cause the substitution of glycine at codon 12 to serine (p.G12S) in one allele of *HRAS* has been observed in 80% of Costello syndrome patients. The G12S mutation, which has been identified in somatic cancer, is an oncogenic mutation that activates the downstream pathway. Patients with Costello syndrome develop a variety of skin abnormalities, including palmoplantar keratoderma, acanthosis nigricans, eczema, loose skin (cutis laxa), darker skin color, and papillomata around nose and anus. However, the pathogenesis of dermatological abnormalities remains unclear.

We have recently generated a strain of knock-in mice expressing an *Hras* G12S mutation, the most frequent mutation in Costello syndrome^12^, which exhibited facial dysmorphia, cardiomyocyte hypertrophy, and kidney anomalies. Impaired mitochondrial fatty acid oxidation was observed in *Hras*^*G12S/+*^ mice fed a high-fat diet^13^. Skin abnormalities, including papillomas, have not been observed in young *Hras*^*G12S/+*^ mice (<30 weeks old) under specific pathogen-free conditions. In contrast, *Hras*^*G12S/+*^ mice over 30 weeks of age or high-fat diet fed-*Hras*^*G12S/+*^ mice had cutaneous lesions due to scratching (Supplementary Fig. 1a) under the same pathogenic-free condition. Although we have not analyzed the histology of skin of *Hras*^*G12S/+*^ mice over 30 weeks of age, gross appearances of the skin lesions and scratching behavior suggest that they are atopic dermatitis-like. In the current study, we tested to generate experimentally induced dermatitis in *Hras*^*G12S/+*^ mice and found that *Hras*^*G12S/+*^ mice developed more severe atopic dermatitis (AD)-like lesions than *Hras*^+/+^ mice after treatment with house dust mite allergens (*Dermatophagoides farinae*, Dfb). Furthermore, these AD-like skin lesions in *Hras*^*G12S/+*^ mice were reversed by treatment with an MEK inhibitor, PD0325901.

## Results

### Dfb ointment induces AD-like skin lesions in *Hras*^*G12S/+*^ mice

**Fig. 1:**
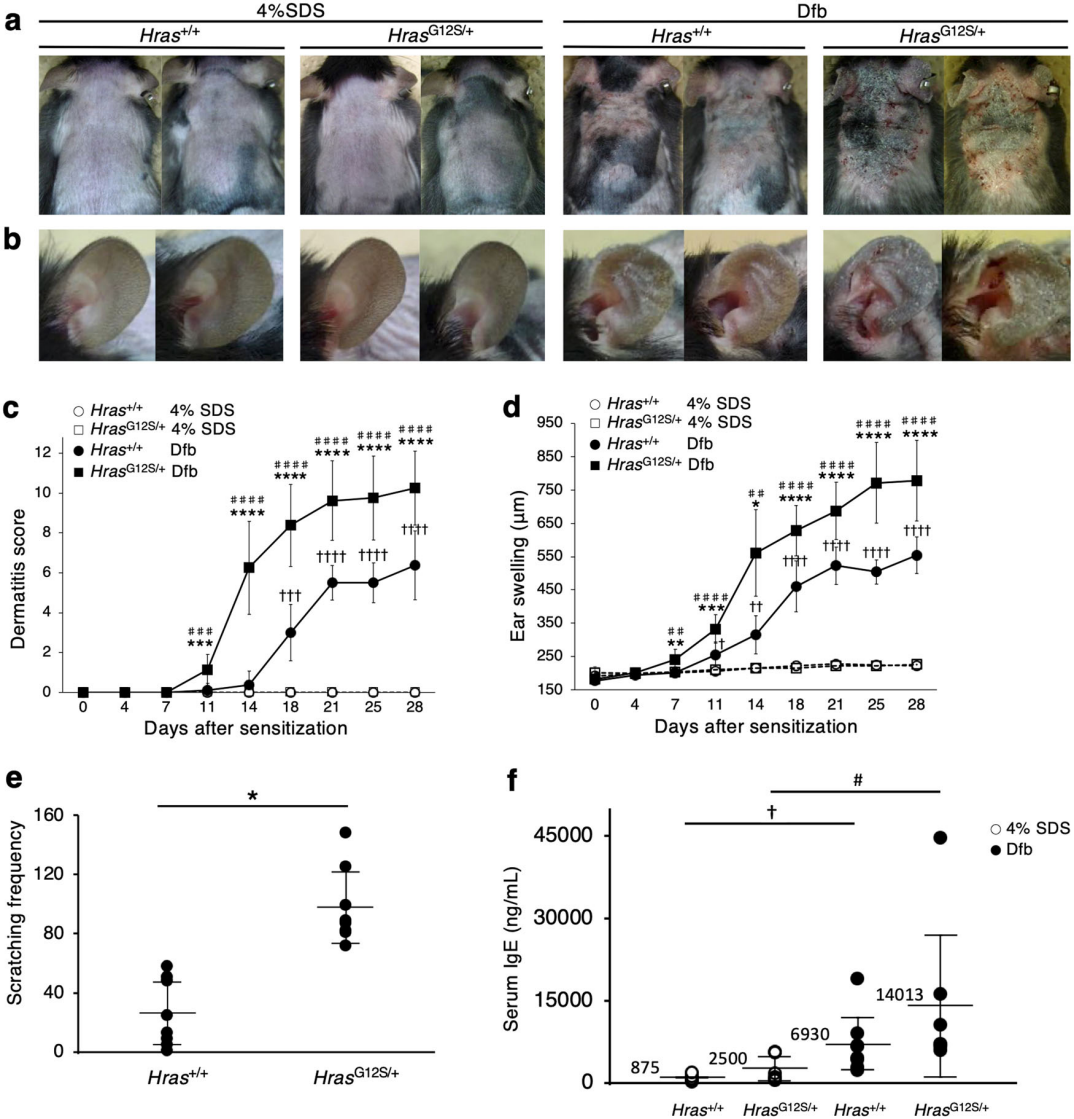
Dfb treatment induces atopic dermatitis-like skin lesions in *Hras*^*G12S/+*^ mice. **a**, **b** Skin and ear features of mice on day 28 after treatment of Dfb ointment. *Hra*s^*G12S/+*^ mice showed dermatitis by repeated application of Dfb (**a**). The severity of ear swelling responses to Dfb was stronger in *Hras*^*G12S/+*^ than *Hras*^*+/+*^ mice (**b**). **c** Dermatitis scores of only 4% SDS-treated (control) *Hras*^*+/+*^ and *Hra*s^*G12S/+*^, and Dfb-treated *Hras*^*+/+*^ and *Hra*s^*G12S/+*^ mice (*n* = 8 per group). **d** Time course of ear thickness from *Hras*^*+/+*^ and *Hras*^*G12S/+*^ mice after treatment with 4% SDS or Dfb (*n* = 8 per group). **e** The number of scratching bouts per 30 min assessed by the video (*n* = 8 per group). **f** Serum IgE levels in *Hras*^*+/+*^ and *Hras*^*G12S/+*^ mice after treatment with 4% SDS or Dfb (*n* = 6 per group). Data are presented as mean ± SD. Significance in (**c**), (**d**), and (**f**) was analyzed by one-way ANOVA and the Tukey−Kramer method. *P < 0.05, **P < 0.001, ***P < 0.001, and ****P < 0.0001 *Hras*^*G12S/+*^ Dfb vs *Hras*^+/+^ Dfb; ^#^*P* < 0.05, ^##^*P* < 0.01, ^###^*P* < 0.001, and ^####^*P* < 0.0001 *Hras*^*G12S/+*^ Dfb vs *Hras*^*G12S/+*^ 4% SDS; ^†^*P* < 0.05, ^††^*P* < 0.01, ^†††^*P* < 0.001, and ^††††^*P* < 0.0001 *Hras*^+/+^ Dfb vs *Hras*^+/+^ 4% SDS. Significance in (**e**) was analyzed by two-tailed Student’s *t* test, *P < 0.05.

We first tested the effect of picryl chloride, which induce contact dermatitis, and imiquimod, which induce psoriasis on the skin of *Hras*^*+/+*^ and *Hras*^*G12S/+*^ mice (Supplementary Fig. 1b, c), but no difference in skin lesions was observed between them (Supplementary Fig. 1d). In contrast, the treatment with Dfb ointment developed severe dermatitis, including severe erythema, hemorrhage, scarring, and eczema, in the dorsal skin of *Hras*^*G12S/+*^ mice, but not in *Hras*^*+/+*^ mice (Fig. 1a and Supplementary Fig. 2a). The ears of *Hras*^*G12S/+*^ mice became thick with edema, erosion, and excoriation (Fig. 1b). The dermatitis score was significantly higher in Dfb-treated *Hras*^*G12S/+*^ mice than in any other group of mice (4% SDS-treated control *Hras*^*+/+*^ mice, Dfb-treated *Hras*^*+/+*^ mice, and 4% SDS-treated control *Hras*^*G12S/+*^ mice) on day 11 (Fig. 1c and Supplementary Table 1). Other dermatitis parameters, including the ear swelling (Fig. 1d) and the scratching behavior (Fig. 1e), increased significantly in Dfb-treated *Hras*^*G12S/+*^ mice compared with Dfb-treated *Hras*^*+/+*^ mice. Serum IgE levels were significantly higher in *Hras*^*G12S/+*^ mice compared to *Hras*^+/+^ in nontreated baseline (261 ± 152.2 ng/mL vs 654 ± 348 ng/mL, *P* = 0.039, Supplementary Fig. 3). Although the difference was not statistically significant, in 4% SDS treatment groups, IgE levels were higher in *Hras*^*G12S/+*^ mice compared to *Hras*^+/+^ (875 ± 596 ng/mL vs 2500 ± 2412 ng/mL, *P* = 0.164, Fig. 1f) as well as in the Dfb treatment groups (6930 ± 5348 ng/mL vs 14,013 ± 13,951 ng/mL, *P* = 0.243, Fig. 1f). These symptoms were also seen in *Hras*^+/+^ mice, but skin lesions are more severe in *Hras*^G12/+^ mice. In both groups of mice, the IgE elevations were triggered by Dfb ointment.

We next examined if Dfb-induced dermatitis in *Hras*^*G12S/+*^ mice is caused by the same pathology as in *Hras*^+/+^ mice.

**Fig. 2:**
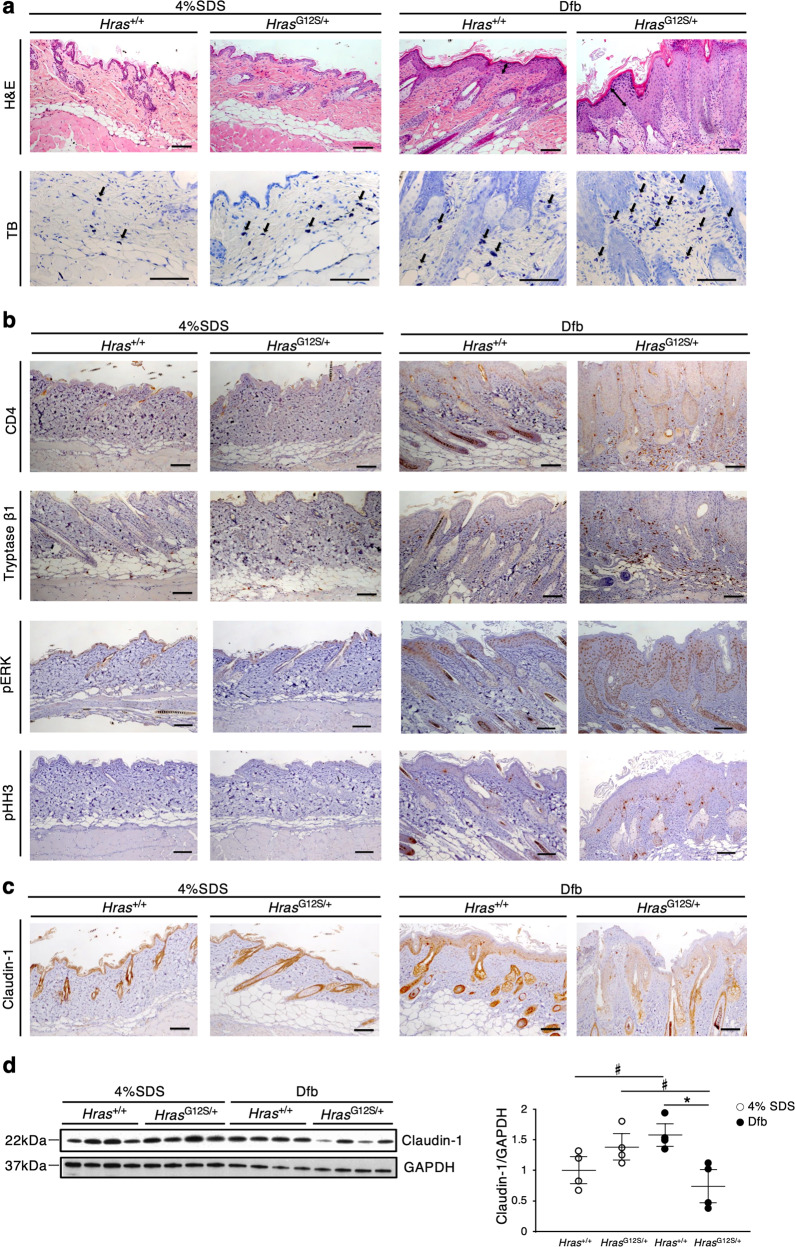
Histological analysis reveals acanthosis with hyperproliferation of p-ERK-positive epidermal cells, increased inflammatory cells, and reduced claudin-1 expression in the dorsal skin of Dfb-treated *Hras*^*G12S/+*^ mice. **a** Skin tissue stained with H&E and TB. **b**, **c** Immunohistochemistry of CD4, tryptase β1, p-ERK, pHH3, and claudin-1 in the skin. **a**–**c** Scale bars: 100 μm. **d** Lysates from the skin were immunoblotted with anti-Claudin-1 antibody. Band intensities were quantified and compared among the four groups. The expression levels were normalized to GAPDH (*n* = 4 in each group). Data are presented as mean ± SD. Significance was analyzed by one-way ANOVA and the Tukey−Kramer method. *P < 0.05, ^#^*P* < 0.05, two-tailed Student’s *t* test.

Histological analysis revealed hyperkeratosis and epidermal hyperplasia in the dorsal skin of Dfb-treated *Hras*^*G12S/+*^ mice (Fig. 2a). The epidermis of *Hras*^*G12S/+*^ mice became thicker than that of *Hras*^*+/+*^ mice, although Dfb treatment increased the epidermal thickness in both *Hras*^*+/+*^ and *Hras*^*G12S/+*^ mice (Supplementary Fig. 2b). In the AD-like skin lesions, Dfb-treated *Hras*^*G12S/+*^ mice displayed increased number of mast cells (toluidine blue^+^ and tryptase β1^+^), a marked increase in the numbers of T cells (CD4^+^) and dendritic cells (MHC class II^+^) (Fig. 2a, b and Supplementary Fig. 2c, e). Western blotting analysis revealed that the levels of CD4 protein were significantly increased in Dfb-treated mice compared with control mice (Supplementary Fig. 2d). In line with the acanthosis of Dfb-treated *Hras*^*G12S/+*^ mice, an increased number of phosphohistone H3-positive cells were observed in the suprabasal epidermis layers of *Hras*^*G12S/+*^ mice (Fig. 2b). Although phosphorylated ERK (p-ERK)-positive cells were also increased in the epidermis of Dfb-treated *Hras*^*+/+*^ and *Hras*^*G12S/+*^ mice, the immunostained area in *Hras*^*G12S/+*^ mice was significantly larger than that in *Hras*^*+/+*^ mice. (Fig. 2b and Supplementary Fig. 2f, g). In addition, we examined the expression of filaggrin and claudin-1 as epidermal barrier markers in AD^14–16^. A decreased expression of claudin-1 was observed in Dfb-treated *Hras*^*G12S/+*^ mice compared with control *Hras*^*G12S/+*^ mice (Fig. 2c, d). Together, these results indicate that Dfb-applied *Hras*^*G12S/+*^ mice exhibited more severe AD-like skin lesions than *Hras*^*+/+*^ mice, including acanthosis with hyperproliferation of p-ERK-positive cells in the epidermis, as well as increased inflammatory cells and reduced claudin-1 expression.

### The skin of Dfb-applied *Hras*^*G12S/+*^ mice shows an increase of itch-associated factors and inflammatory cytokines

**Fig. 3:**
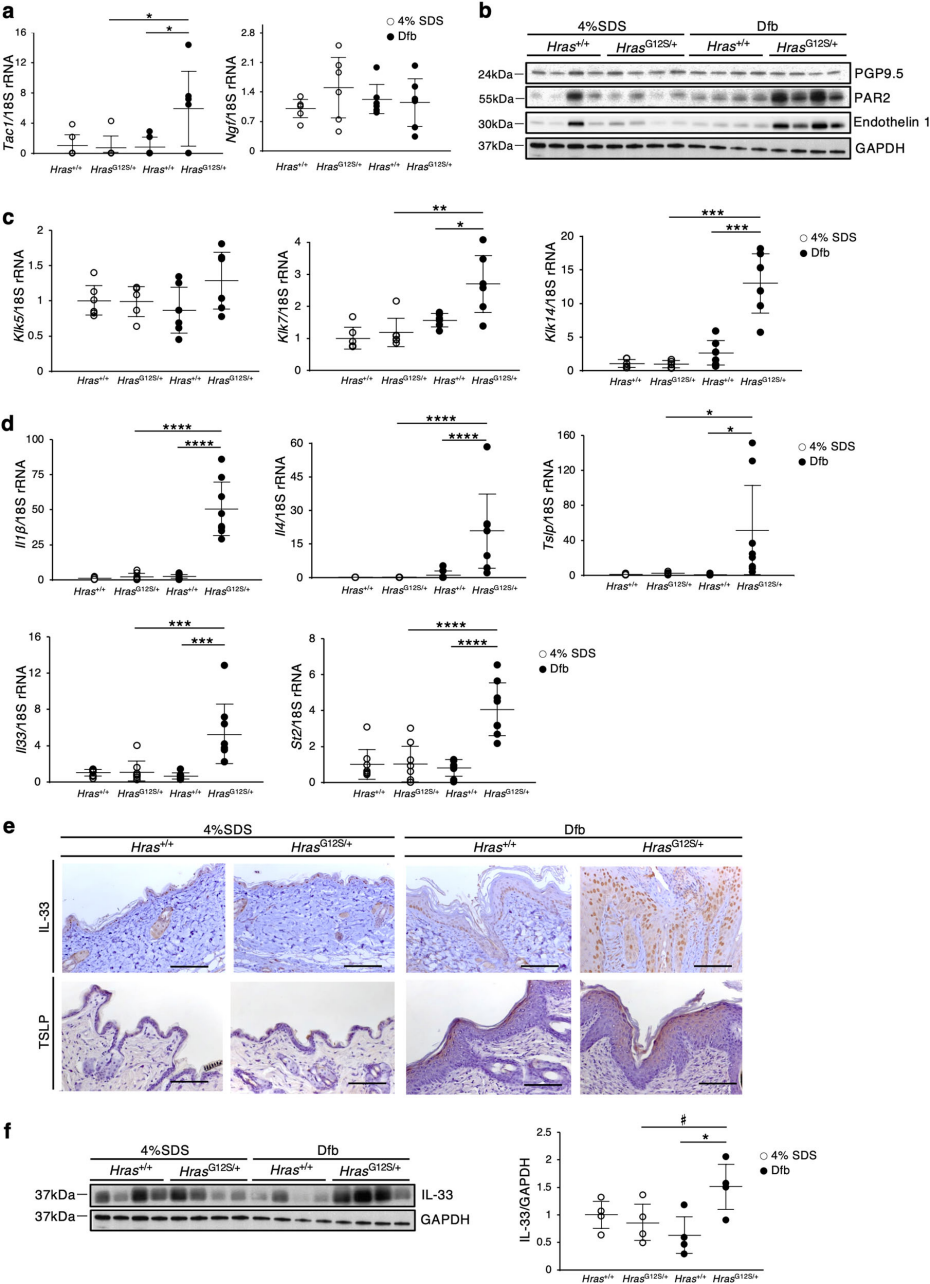
Expression of itch-associated factors and inflammatory cytokines is enhanced in Dfb-induced skin lesions in Hras^*G12S/+*^ mice. **a**, **c**, **d** Relative mRNA expression related to neuronal factors (**a**: *Tac1* and *Ngf*), skin proteases (**c**: *Klk5*, *Klk7*, and *Klk14*), and inflammation (**d**: *Il1β*, *Il4*, *Tslp*, *Il33*, and *St2*) in the dorsal skin. mRNA levels were normalized to those of 18s rRNA (*n* = 8 per group). **b**, **f** Protein extracts from dorsal skin were immunoblotted with anti-PGP9.5, anti-PAR2, anti-Endothelin 1, and anti-IL-33 antibody (*n* = 4 in each group). GAPDH, same data as in Fig. 2d. **f** Band intensities were quantified and compared among the four groups. Expression levels were normalized to GAPDH. **e** Immunohistochemistry of IL-33 and TSLP. Scale bars: 100 μm. Data are presented as mean ± SD. Significance was analyzed by one-way ANOVA and the Tukey−Kramer method. *P < 0.05, **P < 0.01, ***P < 0.001, and ****P < 0.0001. ^#^*P* < 0.05, two-tailed Student’s *t* test.

To further characterize the AD-like skin lesions, we evaluated the levels of the itch-associated factors and inflammatory cytokines in the skin of Dfb-treated *Hras*^*G12S/+*^ mice. Itch-related neuronal markers, including skin *Tac1*, *Klk7* and *Klk14*, mRNA levels or PAR2, and Endothelin 1 proteins^17–20^, were significantly higher than those in control *Hras*^*G12S/+*^ and Dfb-treated *Hras*^*+/+*^ mice (Fig. 3a–c). Regarding inflammatory cytokines, the skin mRNA levels of *Il1β* (proinflammatory cytokine), *Il4* (Th2-related cytokine), and epidermal-derived cytokines, *Il33* and thymic stromal lymphopoietin (*Tslp*), were significantly elevated in Dfb-treated *Hras*^*G12S/+*^ mice compared with control *Hras*^*G12S/+*^ and Dfb-treated *Hras*^*+/+*^ mice (Fig. 3d). IL-33 leads to the activation of type-2 innate lymphoid cells (ILC2s) through ST2, IL-33 receptor^21^. In Dfb-treated *Hras*^*G12S/+*^ mice, the skin mRNA levels of *St2* were also significantly increased (Fig. 3d). Immunohistochemistry analysis revealed that Dfb-treated *Hras*^*G12S/+*^ mice showed enhanced expression levels of IL-33 and TSLP in the basal epidermal layers and the surface of epidermis, respectively (Fig. 3e). Likewise, the IL-33 protein levels were significantly higher in the skin of Dfb-treated *Hras*^*G12S/+*^ mice than in Dfb-treated *Hras*^*+/+*^ mice (Fig. 3f).

### Increased numbers of ILC2 and increased IL-33 expression were observed in the epidermis of *Hras*^*G12S/+*^ mice

**Fig. 4:**
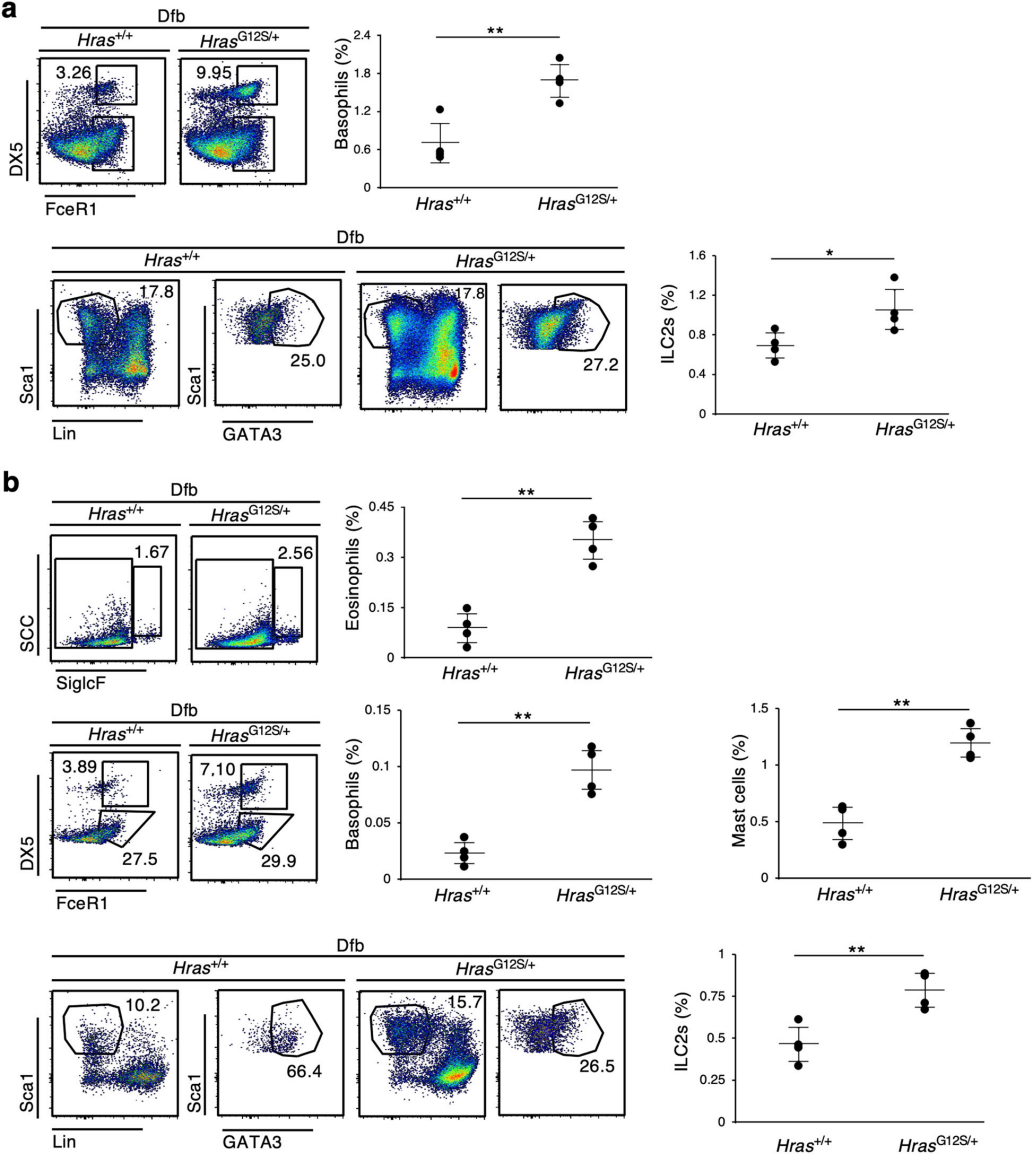
An increase in ILC2s and basophils observed in the skin and ear of Dfb-treated *Hras*^*G12S/+*^ mice. **a**, **b** Flow cytometric analysis of skin (**a**) and ear (**b**) cells from *Hras*^+/+^ and *Hras*^*G12S/+*^ mice collected 12 days after Dfb application. Eosinophils: CD45^+^ SSC^hi^, siglecF^+^, Basophils: CD45^+^ siglecF^−^ FcεRIα^+^ DX5^+^, Mast cells: CD45^+^ siglecF^−^ FcεRIα^+^ DX5^−^, ILC2; CD45^+^ Lin^−^ (CD3ε, CD4, CD8a, CD11c, FcεRIα, NK1.1, CD19, Ter119, CD5, F4/80, Gr-1) Sca1^+^ GATA3^+^ (*n* = 4 in each group). Data are presented as mean ± SD. Significance was analyzed by two-tailed Student’s *t* test. *P < 0.05, **P < 0.01.

**Fig. 5:**
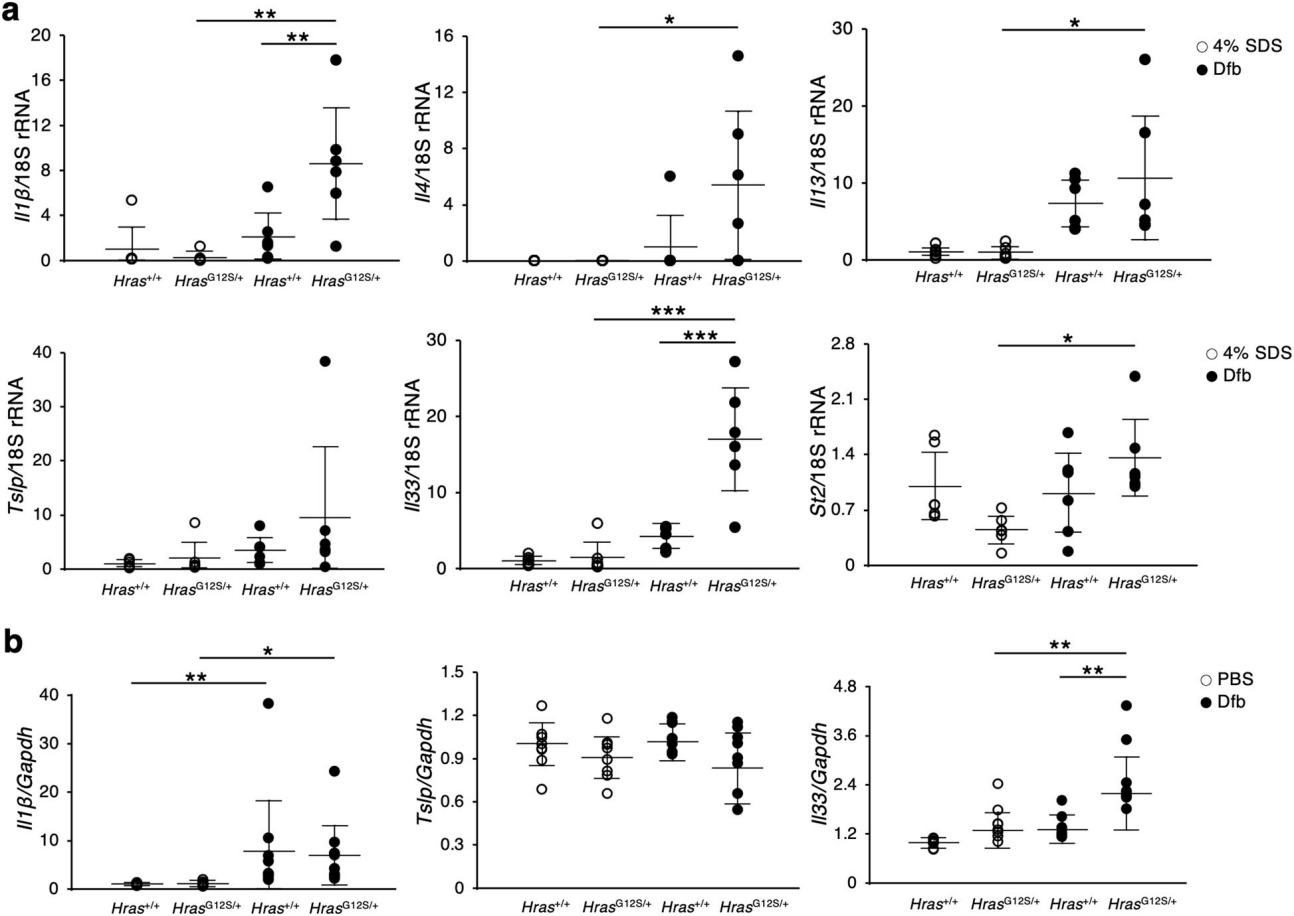
Expression of inflammatory cytokines is enhanced in the skin of Dfb-treated *Hras*^*G12S/+*^ mice in the early stage of dermatitis. **a** Relative mRNA expression related to inflammation, including *Il1β*, *Il4*, *Il13*, *Tslp*, *Il33*, and *St2* in the dorsal skin of *Hras*^+/+^ and *Hras*^*G12S/+*^ mice on day 15 after treatment of Dfb. mRNA levels were normalized to those of 18s rRNA (*n* = 6 per group). **b** Relative mRNA expression of *Il1β*, *Tslp*, and *Il33* in Dfb-stimulated (100 ng/μl, 6 h) or vehicle-treated (PBS, 6 h) keratinocyte from *Hras*^+/+^ and *Hras*^*G12S/+*^ mice. mRNA levels were normalized to those of *Gapdh*. Results represent five independent experiments. Data are presented as mean ± SD. Significance was analyzed by one-way ANOVA and the Tukey−Kramer method. *P < 0.05, **P < 0.01, and ***P < 0.001.

To investigate when and how inflammatory cytokines are induced in *Hras*^*G12S/+*^ mice, we performed flow cytometry on skin and ear samples 12 days after Dfb application, that is, once the AD-like skin lesions had begun to appear (Fig. 1c). The accumulation of basophils and ILC2s was observed on skin samples of Dfb-treated *Hras*^*G12S/+*^ mice 12 days after Dfb application (Fig. 4a). A significant increase in mast cells, eosinophils, basophils, and ILC2s was also observed in the ears of *Hras*^*G12S/+*^ mice (Fig. 4b). On the other hand, these immune cells were hardly detected in *Hras*^*+/+*^ and *Hras*^*G12S/+*^ mice 12 days after 4% SDS application (Supplementary Fig. 4). The mRNA levels of inflammatory cytokines in the skin from *Hras*^*G12S/+*^ mice 15 days after Dfb application showed that *Il1β* and *Il33* were significantly elevated in the skin of Dfb-treated *Hras*^*G12S/+*^ mice compared with Dfb-treated *Hras*^*+/+*^ mice (Fig. 5a).

**Fig. 6:**
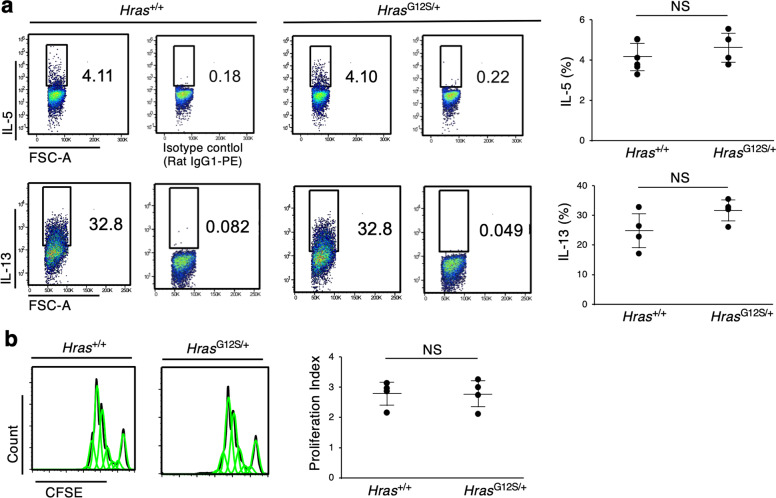
No significant difference was found in Th2 cytokine responses and the proliferation of naive CD4^+^ T cells between *Hras*^*+/+*^ and *Hras*^*G12S/+*^ mice. **a** Naive CD4^+^ T cells were isolated from the spleen of *Hras*^+/+^ and *Hras*^*G12S/+*^ mice at 7 weeks of age. After that, the cells were stained with CSFE and cultured with mouse anti-CD3 monoclonal, anti-CD28 monoclonal, recombinant mouse IL-2, recombinant mouse IIL-4, and anti-IFNγ antibodies for 5 days. After incubation, the cells were fixed, permeabilized, and stained with IL-5 and IL-13 antibodies. Flow cytometric analysis was performed (*n* = 4 in each group). **b** After 3 days culture of purified naive T cells from spleen of *Hras*^+/+^ and *Hras*^*G12S/+*^ mice, the proliferation index was measured by flow cytometry (*n* = 4 in each group). Data are presented as mean ± SD. Significance was analyzed by two-tailed Student’s *t* test.

Atopic dermatitis is characterized by increased serum IgE, acanthosis, loss of skin barrier function, and infiltration of immune cells, including Th2 cells, dendritic cells, eosinophils, basophils, and mast cells^22,23^. On that account, we next examined the response of immune cells in these mice. HRAS is known to be highly expressed in the epidermal cells, but not in immune cells^24,25^. Indeed, no significant difference was found in the population of immune cells from the spleen and lymph node (LN). The proliferation of naive CD4^+^ T cells and Th2 immune response was comparable between *Hras*^*+/+*^ and *Hras*^*G12S/+*^ mice at 7 weeks of age, suggesting that AD-like dermatitis may not be caused by different response of immune cells (Fig. 6a, b). Then we examined whether an increased production of IL-33 and TSLP was observed in primary epidermal keratinocytes in response to Dfb. Six hours after Dfb stimulation, the mRNA level of *Il1β*, not *Tslp*, was significantly elevated in cultured epidermal keratinocytes of *Hras*^*+/+*^ and *Hras*^*G12S/+*^ mice (Fig. 5b). Notably, the mRNA levels of *Il33* in the Dfb-stimulated keratinocytes of *Hras*^*G12S/+*^ mice were significantly increased compared with those of nonstimulated *Hras*^*G12S/+*^ and Dfb-stimulated *Hras*^*+/+*^ keratinocytes, suggesting that epidermal keratinocytes with *Hras* G12S mutation have increased IL-33 expression after stimulation with Dfb (Fig. 5b).

### PD0325901 reduces skin lesions in Dfb-stimulated *Hras*^*G12S/+*^ mice

**Fig. 7:**
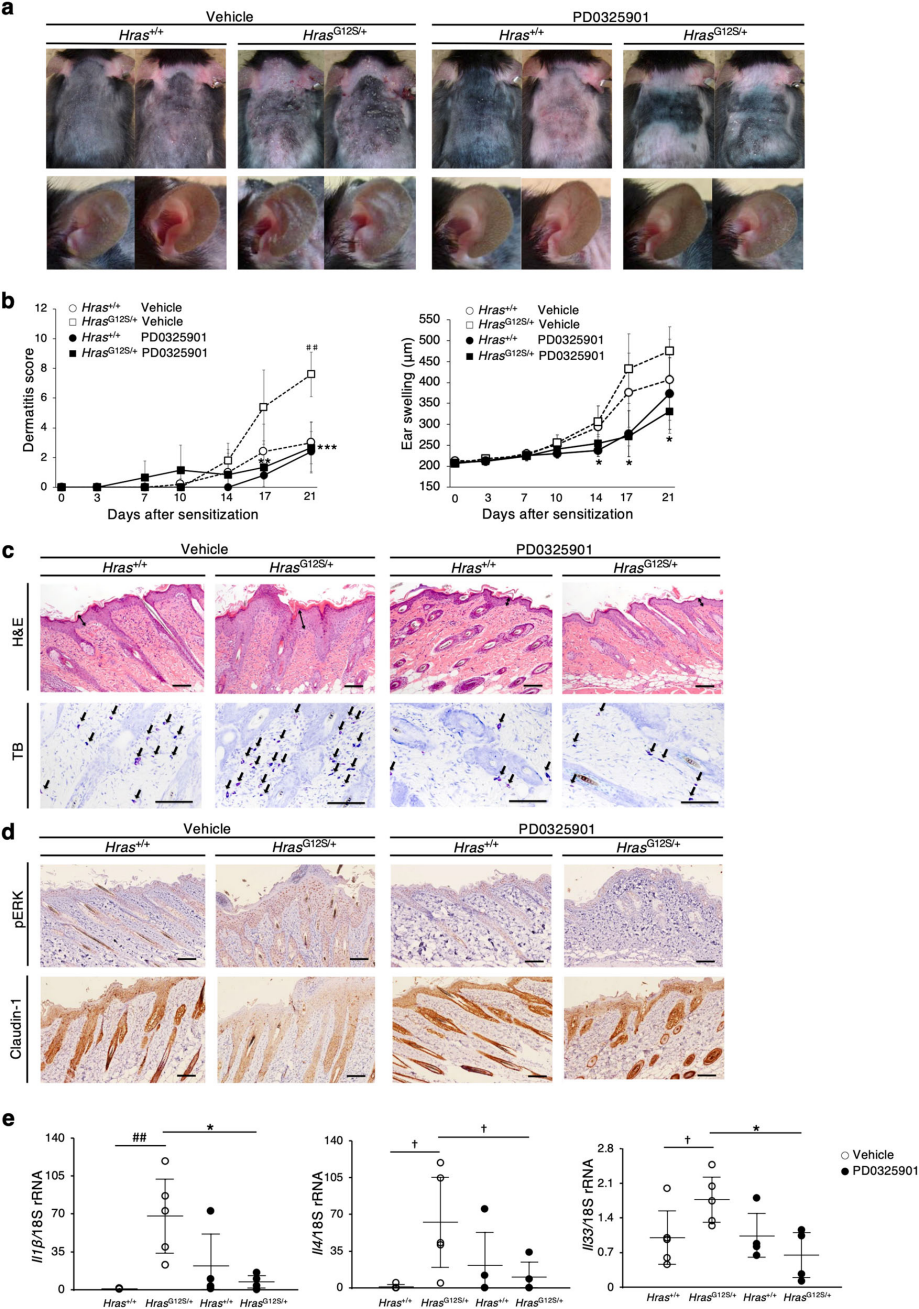
MEK inhibitor reduces the clinical severity of dermatitis in Dfb-stimulated *Hras*^*G12S/+*^ mice. **a**, **b** Gross appearances, dermatitis score, and ear thickness in vehicle (saline) or PD0325901-treated *Hras*^+/+^ and *Hras*^*G12S/+*^ mice after 10 days of daily injections (*n* = 5 per group). All of the mice in these figures were treated with Dfb. The open circle and open square show vehicle group (without MEKi, with same amount of saline). **c** Skin tissue stained with H&E and TB in *Hras*^+/+^ and *Hras*^*G12S/+*^ mice with PD0325901 or vehicle treatment (*n* = 5 per group). **d** Immunohistochemistry of p-ERK and claudin-1 in skin. **c**, **d** Scale bars: 100 μm. **e** Relative mRNA expression of *Il1β*, *Il4*, and *Il33* in dorsal skin. mRNA levels were normalized to those of 18s rRNA (vehicle group: *n* = 5; PD0325901 group: *n* = 4). Data are presented as mean ± SD. Significance was analyzed by one-way ANOVA and the Tukey−Kramer method. *P < 0.05, **P < 0.01, and ***P < 0.001 *Hras*^*G12S/+*^ PD0325901 vs *Hras*^*G12S/+*^ vehicle; ^##^*P* < 0.01, *Hras*^*G12S/+*^ vehicle vs *Hras*^+/+^ vehicle. ^†^*P* < 0.05, one-tailed Student’s *t* test.

Treatment with MEK inhibitors ameliorates the abnormalities observed in RASopathy-related model mice^26–28^. We investigated the effects of an MEK inhibitor, PD0325901, on skin lesions in Dfb-treated *Hras*^*G12S/+*^ mice (Supplementary Fig. 5a). Ten days of treatment with PD0325901 (or saline) resulted in a significant improvement of the dermatitis score and ear swelling in *Hras*^*G12S/+*^ mice (Fig. 7a, b). PD0325901 treatment resulted in a marked reduction of epidermal thickness, mast cell numbers, and p-ERK-positive epidermal cells, as well as recovered expression levels of claudin-1 (Fig. 7c, d and Supplementary Fig. 5b, c). The mRNA levels of *Il1β*, *Il4, Il33*, *St2*, and *Klk14* were significantly lower in the skin of PD0325901-treated *Hras*^*G12S/+*^ mice than that of vehicle (saline)-treated *Hras*^*G12S/+*^ mice (Fig. 7e and Supplementary Fig. 5d). These results suggest that ERK inhibition partially ameliorates Dfb-induced skin lesions in *Hras*^*G12S/+*^ mice.

## Discussion

**Fig. 8:**
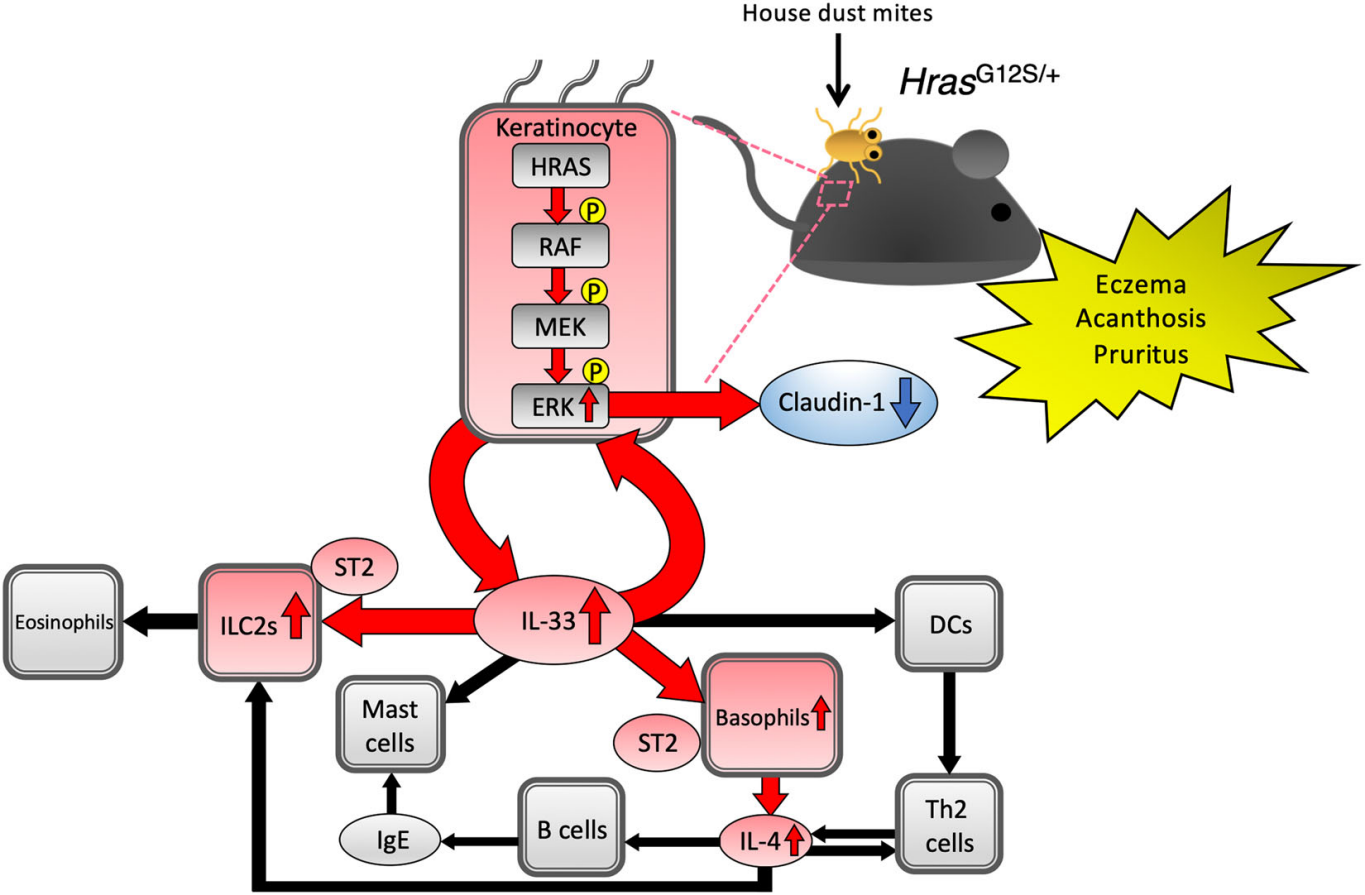
Germline *Hras*^*G12S*^ mutation causes AD-like skin lesions via ERK/IL-33 axis. Exposure to Dfb allergen induces AD-like skin lesions, including eczema, acanthosis, and pruritus in *Hras*^*G12S/+*^ mice. Dfb-treated *Hras*^*G12S/+*^ keratinocytes show increased IL-33 expression through hyperproliferation of p-ERK-positive epidermal cells. Excess IL-33 activates basophil- and ILC2-containing ST2 receptors. These activated immune cells induce the production of type-2 inflammatory cytokines, such as IL-4. Furthermore, excess IL-33 can activate ERK signaling, resulting in reduced claudin-1 expression and skin barrier dysfunction. DC dendritic cells.

Here, we demonstrated that mice expressing a germline *Hras* G12S mutation, but not *Hras*^*+/+*^ mice, developed AD-like skin lesions under conditions of Dfb exposure. The levels of IL-1β and epithelial cell-derived cytokines, IL-33 and TSLP, were also increased in Dfb-treated *Hras*^*G12S/+*^ mice. In addition, an increased production of IL-1β, IL-4, and IL-33, as well as inflammatory cells, basophils, and ILC2s, was observed in the dorsal skin of *Hras*^*G12S/+*^ mice before the development of AD-like skin lesions. Analysis of the underlying mechanism revealed that Dfb-stimulated keratinocytes in *Hras*^*G12S/+*^ mice induced IL-33 production, while, in naive CD4^+^ T cells from the spleen, the Th2 immune response was comparable between *Hras*^*+/+*^ and *Hras*^*G12S/+*^ mice. Finally, the inhibition of ERK activation by PD0325901 treatment ameliorated the AD-like skin lesions and IL-33 production. Together, these data indicate that germline *Hras* G12S activating mutation causes AD-like skin lesions via the ERK/IL-33 axis (Fig. 8).

In the present study, after repeated stimulation with Dfb, *Hras*^*G12S/+*^ mice showed hyperproliferation of p-ERK-positive epidermal cells and increased IL-33 expression in the dorsal skin. Increased IL-33 expression was also observed in primary epidermal keratinocytes from *Hras*^*G12S/+*^ mice after Dfb stimulation, which is similar to the reports that the activation of RAS/MAPK signaling was associated with increased IL-33 expression in cancer cells^29,30^. Recently, IL-33 was found to induce the Th2 inflammatory response in allergic diseases, especially AD^31^. Excess IL-33 is also associated with skin barrier dysfunction and ILC2 functions, which is partially regulated by the RAS/MAPK signaling pathway^32,33^. Epithelial-specific IL-33 transgenic mice have been found to develop AD-like dermatitis, including acanthosis, pruritus, increased IgE serum levels, reduced claudin-1 expression, and increased production of eosinophils, mast cells, and ILCs^33,34^. Of note, IL-33 and its receptor, ST-2, are highly expressed in the skin-derived ILC2s of AD patients and lung ILC2s of patients with allergic airway diseases, respectively^35,36^. Importantly, consistent with the phenotype of IL-33 transgenic mice and AD patients, Dfb-treated *Hras*^*G12S/+*^ mice showed AD-like skin lesions, reduced claudin-1 expression, increased IL-33 expression, hyperproliferation of p-ERK-positive epidermal cells, and increased ILC2 production. Collectively, excess IL-33 could lead to ERK activation, resulting in an increase of ILC2s and impaired skin barrier (Fig. 8).

The pathogenesis of skin lesions observed in Costello syndrome includes dermal connective tissue abnormalities (cutis laxa and deep palmer and plantar creases), hyperproliferative skin disease (palmoplantar keratoderma, cutaneous papilloma, and acanthosis nigricans), and inflammatory skin abnormalities (sensitive skin, eczema, and pruritus). A previous study showed that, in the skin fibroblasts of Costello syndrome, elastic fibers were not assembled due to a functional deficiency of the elastin-binding protein as a result of an unusual accumulation of chondroitin sulfate-bearing proteoglycans^37,38^. Regarding the hyperproliferative skin lesions, it has been reported that the root cause of papillomas, hyperkeratosis, and epidermal hyperplasia, such as psoriasis, is the activation of the RAS-MAPK pathway^39–42^. However, the pathophysiological mechanism of inflammatory skin abnormalities in Costello syndrome remains unclear. In the present study, Dfb-treated *Hras*^*G12S/+*^ mice displayed pruritus and eczema. Recently, mice with epidermis-specific BRAF/RAF1 deficient also showed AD-like dermatitis, which is characterized by increased serum IgE levels and a Th2 response^43^. The elevated IgE levels have not been systematically examined or reported in Costello syndrome patients. However, it is possible that the allergic reaction stimulated by house dust mites could be involved in the development of inflammatory skin abnormalities in Costello syndrome patients, including sensitive skin, pruritus, and eczema.

At present, acitretin has been reported to treat palmoplantar keratosis in patients with Costello and CFC syndrome^44,45^. Several reports have demonstrated that MEK inhibitors improve HRAS-driven tumorigenesis^46^, impaired enamel formation in the teeth^27^, and long-term depression in the hippocampus in *Hras* G12V knock-in mice^28^, as well as hyperkeratosis and hyperplasia in the forestomach of *Braf*^*Q241R/+*^ mice^26^. In the present study, PD0325901 treatment of the AD-like skin lesions in *Hras*^*G12S/+*^ mice was found to reverse these lesions by reducing hyperproliferation of p-ERK-positive epidermal cells and the production of inflammatory cells and cytokines, including IL-1β, IL-4, and IL-33. Treatment with U0126, an MEK inhibitor, in human keratinocytes, has also been found to restore the reduced expression levels of claudin-1 and filaggrin, and increase ERK activation through excess IL-33^33,47^. Indeed, reduced claudin-1 expression was improved in Dfb-treated *Hras*^*G12S/+*^ mice after PD0325901 treatment. Recently, hypertrophic cardiomyopathy in Noonan syndrome patients were treated by MEK inhibitor^48^. So, MEK inhibitors could be effective in patients with RASopathies. The most common side effect of trametinib (MEK inhibitor) in human patients is skin rush, and common toxicity associated with vemurafenib (BRAF inhibitor) is cutaneous abnormalities such as keratoacanthoma and squamous cell carcinoma by the mechanism of paradoxical MAPK pathway activation^49^. Therefore, the balance of RAS/MAPK signaling plays an important role in the emersion of skin abnormalities. Adjusting dosage of MEK inhibitors may be effective on skin lesions of patients with Costello syndrome.

Here, Dfb-treated *Hras*^*G12S/+*^ mice exhibited increased IL-33 expression through hyperproliferation of p-ERK-positive epidermal cells. Additionally, we show that PD0325901 treatment ameliorated the AD-like skin lesions in *Hras*^*G12S/+*^ mice under conditions of exposure to Dfb. Thus, it will be interesting to investigate whether treatment with IL-33 antibody reduces the AD-like skin lesions in *Hras*^*G12S/+*^ mice. Our findings provide additional perspective that *Hras*^G12S/+^ mice will serve as a valuable model to study pathophysiology and potential therapeutic approaches in AD.

## Materials and methods

### Mice

*Hras*^*G12S/+*^ mice on a C57BL/6J background have been described previously^13^. Male mice were analyzed in this study.

### Genotyping

The genomic DNA was extracted from the tail tissue using a Maxwell 16 Mouse Tail DNA Purification Kit (Promega, Madison, WI, USA) or the alkaline lysis method. For the alkaline lysis method, a small piece of each tail (2 mm) was incubated in 50 mM NaOH for 20 min at 95 °C. After the addition of 1 M Tris-HCl (pH 8.0), the extracts were used for PCR. Genotyping of *Hras*^+/+^ and *Hras*^*G12S/+*^ mice was performed by PCR using KOD FX Neo (TOYOBO, Osaka, Japan). The primers used for PCR have been described previously^13,50^.

### Induction of dermatitis

Atopic dermatitis-like skin lesions were induced in male mice at 9 weeks of age, according to the manufacturer’s instructions. The mice were anesthetized with isoflurane and their dorsal hair was removed using an electric clipper (2000AD; Natsume Seisakusho, Tokyo, Japan). For sensitization, 100 mg of Biostir AD (Biostir Inc, Kobe, Japan), an ointment of Dfb extract, was applied to the shaved dorsal skin and ears. Three or four days later, hair growth was removed with an electric clipper, and 150 μl of 4% SDS (Sigma-Aldrich, St. Louis, MO, USA) was applied to the shaved dorsal skin and ears to disrupt the skin barrier. After 2 h, 100 mg of Biostir AD was applied to their shaved dorsal skin and ears to induce AD-like skin lesions. These procedures were repeated twice a week for 25 days. The mice were sacrificed on day 32 to collect skin and ear samples (Supplementary Fig. 2a). To assess the effect of Dfb ointment to *Hras*^+/+^ and *Hras*^*G12S/+*^ mice, they were randomly divided into four groups (4% SDS-treated *Hras*^+/+^, 4% SDS-treated *Hras*^*G12S/+*^, Dfb-treated *Hras*^+/+^, and Dfb-treated *Hras*^*G12S/+*^) using single blinding test.

### Evaluation of dermatitis and ear thickness

The dermatitis scores were evaluated twice a week according to the development of four symptoms: erythema/hemorrhage of dorsal skin, scarring/dryness of dorsal skin, edema of ear, and excoriation/erosion of ear^51^. The total dermatitis score (maximum score: 12) was defined as the sum of individual scores (none: 0, mild: 1, moderate: 2, severe: 3) for each symptom (Supplementary Table 1). Ear thickness was measured with a digimatic micrometer (CLM1-15QM; Mitutoyo, Kanagawa, Japan).

### Measurement of scratching behavior

On day 28, scratching behavior was monitored by video (GZ-HM890; JVC, Kanagawa, Japan) for 30 min. The number of scratching bouts was assessed by replaying the video. Each incidence of scratching behavior was defined as rubbing of ears and dorsal skin with forepaws, hind paws, and mouth.

### Histology and immunohistochemistry

The dorsal skins were fixed in 10% neutral buffered formalin for paraffin-embedded specimen and 4% paraformaldehyde for frozen specimen. Embedded tissues were sectioned at 3 μm (paraffin-embedded specimens) or 8 μm (frozen specimens). Paraffin-embedded specimens were stained with hematoxylin and eosin (H&E) and toluidine blue (TB). Epidermal thickness was measured in five randomly selected areas (900 × 700 μm) of each H&E-stained sample. Mast cells were counted in ten randomly selected areas (450 × 350 μm) of each TB-stained sample. For immunohistochemistry, the paraffin-embedded sections were deparaffinized using xylene and rehydrated with ethanol. Frozen specimens were dried sufficiently with a dryer. Antigens were activated using a Histofine simple stain kit (Nichirei Bio Sciences, Tokyo, Japan). The antibodies used are described in Supplementary Table 2. Signals were visualized with a DAB Substrate Kit (Nichirei Bio Sciences). Sections were counterstained with hematoxylin. p-ERK immunostained area/epidermis (%) was measured in five randomly selected areas (900 × 700 μm) of each p-ERK-stained sample.

### Quantitative reverse transcription-PCR

Total RNA extraction and purification of keratinocytes was performed according to the standard procedures using an RNeasy Mini Kit (Qiagen, Hilden, Germany) and QIAshredder (Qiagen). The extraction and purification of the total RNA from the dorsal skin and cDNA synthesis were performed as previously described^26^. Quantitative real-time PCR was performed using THUNDERBIRD SYBR qPCR MIX (TOYOBO) in a StepOnePlus (Thermo Fisher Scientific, Waltham, MA, USA). The amplification primers are described in Supplementary Table 3. Each sample was run in duplicate.

### Western blotting

Skin tissue was homogenized in lysis buffer (10 mM Tris-HCl pH 7.5 and 1% SDS) containing phosphatase and protease inhibitor (P5726 and P8340; Sigma-Aldrich). Supernatants were collected after centrifugation and the protein concentration was determined using a Bio-Rad Protein Assay (Bio-Rad Laboratories, Hercules, CA). Western blot analyses were performed as previously described^26^. Briefly, the proteins were transferred onto nitrocellulose membranes and blocked with 5% non-fat milk in Tris-buffered saline with Tween-20 (10 mmol/L Tris-HCl, pH 8.0, 150 mmol/L NaCl, and 0.1% Tween-20) for 1 h at room temperature. The membranes were incubated overnight at 4 °C with the antibodies described in Supplementary Table 2. All membranes were visualized using the Western Lightning ECL Plus Kit (PerkinElmer, Waltham, MA, USA). The band intensities were quantified using NIH ImageJ software.

### ELISA

Serum IgE was determined using LBIS Mouse IgE ELISA Kit (FUJIFILM Wako Shibayagi, Gunma, Japan), according to the manufacturers’ instructions.

### Skin and ear immune cell preparation and flow cytometry

The hair on the dorsal skin was shaved off before removing the subcutaneous fat and connective tissue. The skin and ear were cut into pieces and incubated in RPMI 1640 (FUJIFILM Wako, Osaka, Japan) containing 10% fetal calf serum (FCS), Collagenase (C5138; Sigma-Aldrich), Dispase (17105-04; Gibco BRL, Palo Alto, CA), DNase I, and Hyaluronidase (Sigma-Aldrich) for 90 min to isolate single-cell suspension^52^. After incubation in 2.4G2 (130-092-575; Miltenyi Biotec, Auburn, CA) as an Fc-blocking reagent, cells were stained with the antibodies described in Supplementary Table 2. Flow cytometry data were acquired by Spectral Analyzer (SP6800; SONY, Tokyo, Japan) and analyzed with FlowJo (Becton, Dickinson and Company, USA).

### Spleen and LN immune cell preparation and flow cytometry

The spleen and inguinal LN from mice were cut into pieces and incubated in ACK lysis buffer (150 mM NH_4_Cl, 10 mM KHCO_3_, 0.1 mM ethylenediaminetetraacetic acid 2Na, pH 7.2) for 2 min. After incubation in 2.4G2 (Miltenyi Biotec) as an Fc-blocking reagent, the cells were stained with the antibodies described in Supplementary Table 2. The flow cytometry data were acquired by BD FACSCANTO II (BD Bioscience, Franklin Lakes, New Jersey) and analyzed with FlowJo (Becton).

### Spleen immune cell preparation and naive CD4^+^ T-cell sorting assays

Mouse naive CD4^+^ T cells were isolated from the spleen by magnetic sorting according to standard procedures using a naive CD4^+^ T-cell isolation kit (Miltenyi Biotec). CFSE (C1157; Invitrogen, San Diego, CA)-stained cells were suspended in RPMI 1640 medium containing 10% FCS. The cell suspensions were cultured and stimulated with plate-bound mouse anti-CD3 and anti-CD28 monoclonal antibodies, recombinant mouse IL-2, IL-4, and anti-IFNγ antibodies for 5 days in a 37 °C, 5% CO_2_ incubator. The cell suspensions were re-stimulated with phorbol 12-myristate 13-acetate (Sigma-Aldrich), ionomycin (Sigma-Aldrich), and GolgiPlug (BD Bioscience) for 3 h. Cells were fixed and permeabilized with fixation/permeabilization solution (BD Bioscience) and stained with IL-5 and IL-13 antibodies. After 3 days of culturing the purified naive T cells, the proliferation index was determined by flow cytometry. The flow cytometry data were acquired by Spectral Analyzer (SONY) and analyzed with FlowJo (Becton).

### Cell culture and stimulation

Primary epidermal keratinocytes were isolated from newborn mice (0- or 1-day-old) and cultured according to recommended protocol using a CnT-PR (CELLnTEC, Bern, Switzerland), CnT-GAB10 (CELLnTEC), Dispase II (Gibco BRL), and TrypLE Express Enzyme (Gibco BRL). Briefly, the skin was peeled from the neonates and incubated in CnT-PR with CnT-GAB10 (10 μg/mL gentamycin and 0.5 μg/mL amphotericin B) and 12.5 U/mL dispase for 16 h at 4 °C. The epidermis was separated from the dermis and incubated in TrypLE Express Enzyme for 20–30 min at room temperature. The cells were separated from the epidermal sheet, seeded at a density of 4 × 10^4^ cells/cm^2^ in CnT-PR medium, and cultured in a 37 °C, 5% CO_2_ incubator. Medium change was performed after 24 h and 4 days after seeding. After 5 days of culture, 100 ng/mL of mite dermatophagoides farina crude extract (LSL, Tokyo, Japan) in phosphate buffered saline (PBS) was added to the culture medium. After 6 h of incubation, the cells were collected for quantitative reverse transcription-PCR analysis.

### Mouse treatment

PD0325901 (Sigma-Aldrich) was dissolved in ethanol at a concentration of 5 mg/mL and prepared in saline at a concentration of 0.125 mg/mL. PD0325901 (1.0 mg/kg/body weight) was intraperitoneal injected into anesthetized mice daily from days 12 to 21 after the start of Dfb application (Supplementary Fig. 5a).

### Statistical analysis

All data are presented as mean ± standard deviation (SD). Statistical analyses were performed using Student’s *t* test for comparisons between two groups. Comparisons among four groups were performed using one-way ANOVA and the Tukey−Kramer method. All data were analyzed using JMP Pro 14 software (SAS, Cary, NC). *P* value below 0.05 was considered statistically significant.

### Study approval

All animal studies were approved by the Animal Care and Use Committees of Tohoku University.

## Supplementary information

Supplementary figure legend

Supplementary table

Supplementary Figure 1

Supplementary Figure 2

Supplementary Figure 3

Supplementary Figure 4

Supplementary Figure 5

## Data Availability

The authors declare that all other data of this study are available from the corresponding author upon reasonable request.
