# Supplementary material for: Costello syndrome model mice with a HrasG12S/+ mutation are susceptible to develop house dust mite-induced atopic dermatitis
Source: Cell Death Dis. 2020 Aug 13;11(8):617. doi: 10.1038/s41419-020-02845-8 (PMC7426869; doi:10.1038/s41419-020-02845-8)
Supplement: Supplementary file 1 — Supplementary figure legend [file 41419_2020_2845_MOESM1_ESM.docx]

**Supplementary Materials:**

**Supplementary figure 1. Cutaneous phenotype of *Hras*^+/+^, *Hras*^G12S/+^ mice.** (a) *Hras*^G12S/+^ mice over 30 weeks of age showed cutaneous lesions due to scratching. (b) Experimental procedure for induction of contact dermatitis by application of picryl chloride (PiCl). (c) Experimental procedure for induction of psoriasis-like skin lesions by imiquimod. (d) Treatment with imiquimod induced the same levels of psoriasis-like dermatitis between *Hras*^+/+^ and *Hras*^G12S/+^ mice.

**Supplementary figure 2. *Hras*^G12S/+^ mice show increased epidermal thickness, an increase of mast cells and MHC class II^+^ cells in dorsal skin, and increased CD4 expression after Dfb application.** (a) Experimental procedure for the induction of AD-like skin lesions. (b) Epidermal thickness was measured in 5 randomly selected areas (900 × 700 μm) of each H&E stained sample (*n* = 8 per group). (c) Mast cells were counted in 10 randomly selected areas (450 × 350 μm) of each TB stained sample (*n* = 8 per group). (d) Protein extracts were prepared from dorsal skin of 4% SDS-treated *Hras^+/+^* and *Hra*s*^G12S/+^* mice, and Dfb-treated *Hras^+/+^* and *Hra*s*^G12S/+^* mice and immunoblotted with anti-CD4 antibody (*n* = 4 in each group). Band intensities were quantified and compared among 4 groups. The expression levels ware normalized to GAPDH (same data as in Fig. 2d). (e) Immunohistochemistry analysis of MHC class II in dorsal skin. Scale bars: 100 μm (*n* = 8 per group). (f-g) p-ERK immunostained area / epidermis and field (%) was measured in 5 randomly selected areas (900 × 700 μm) of each pERK stained sample (*n* = 4 per group). Data are presented as mean ± SD. Significance was analyzed by one-way ANOVA and the Tukey-Kramer method. **P < 0.01, ***P < 0.001, and ****P < 0.0001.

**Supplementary figure 3. Total serum IgE levels in non-treated *Hras^G12S/+^* mice are significantly higher than those in non-treated *Hras^+/+^* mice at 9 weeks of age.** Data are presented as mean ± SD (*n* = 8 per group). Significance was analyzed by two-tailed Student’s *t*-test. *P < 0.05.

**Supplementary figure 4. Immune cells in the skin and ear of 4%SDS treated mice.** (a-b) Flow cytometric analysis of skin (a) and ear (b) cells from *Hras*^+/+^ and *Hras*^G12S/+^ mice collected 12 days after 4% SDS application. Eosinophils: CD45 SSC, siglecF, Basophils: CD45 siglecF FcεRIα DX5 , Mast cells: CD45 siglecF FcεRIα DX5, ILC2: CD45 Lin (CD3ε, CD4, CD8a, CD11c, FcεRIα, NK1.1, CD19, Ter119, CD5, F4/80, Gr-1) Sca1+ GATA3+ (*n* = 4 in each group). Data are presented as mean ± SD. Significance was analyzed by two-tailed Student’s *t*-test. NS, not significant.

**Supplementary figure 5. Epidermal thickness, number of mast cells, and mRNA levels of *St2* and *Klk14* in Dfb-treated mice are significantly reduced by PD0325901 treatment.** (a) Experimental procedure for the induction of AD like-skin lesions and PD0325901 therapy. (b) Epidermal thickness was measured in 5 randomly selected areas (900 × 700 μm) of each H&E stained sample (*n* = 5 per group). (c) Mast cells were counted in 10 randomly selected areas (450 × 350 μm) of each TB stained sample (*n* = 5 per group). (d) Relative mRNA expression of *Tslp*, *St2*, and *Klk14* in the dorsal skin. mRNA levels were normalized to those of 18S rRNA (vehicle group: *n* = 5; PD0325901 group: *n* = 4). Data are presented as mean ± SD. Significance was analyzed by one-way ANOVA and the Tukey-Kramer method. *P < 0.05, **P < 0.01, ***P < 0.001, and ****P < 0.0001.
