# Supplementary material for: Costello syndrome model mice with a HrasG12S/+ mutation are susceptible to develop house dust mite-induced atopic dermatitis
Source: Cell Death Dis. 2020 Aug 13;11(8):617. doi: 10.1038/s41419-020-02845-8 (PMC7426869; doi:10.1038/s41419-020-02845-8)
Supplement: Supplementary file 2 — Supplementary table [file 41419_2020_2845_MOESM2_ESM.docx]

**Supplementary Materials:**

**Supplementary Table 1. Dermatitis score**

| **Erythema/Hemorrhage (skin)** | | |
| --- | --- | --- |
| **0** | none |  |
| **1** | mild |  |
| **2** | moderate |  |
| **3** | severe |  |
| **Scaling/Dryness (skin)** | |  |
| **0** | none |  |
| **1** | mild |  |
| **2** | moderate |  |
| **3** | severe |  |
| **Edema (ear)** | |  |
| **0** | none |  |
| **1** | mild |  |
| **2** | moderate |  |
| **3** | severe |  |
| **Excoriation/Erosion (ear)** | | |
| **0** | none |  |
| **1** | mild |  |
| **2** | moderate |  |
| **3** | severe |  |
|  |  |  |
|  |  |  |
|  |  |  |

**Supplementary Table 2. Sources of antibodies used in this study**

| **Primary Antibody**  **(Immunohisto chemistry)** | **Clone number** | **Catalog number** | **Brand** |
| --- | --- | --- | --- |
| CD4 | GK1.5 | MAB554 | R&D SYSTEMS |
| MHC class II | M5/114.15.2 | 14-5321-82 | eBioscience |
| Tryptase β-1 | - | AF1937 | R&D SYSTEMS |
| Claudin-1 | - | LS-B6327 | LSBio |
| IL-33 | - | AF3626 | R&D SYSTEMS |
| TSLP | - | Ab188766 | Abcam |
| pERK (P-p44/42 MAPK) | 20G11 | 4376 | Cell Signaling Technology |
| pHH3 | - | 9701S | Cell Signaling Technology |

| **Primary Antibody (Western blot)** | **Clone number** | **Catalog number** | **Brand** |
| --- | --- | --- | --- |
| GAPDH | D16H11 | 5174S | Cell Signaling Technology |
| CD4 | GK1.5 | MAB554 | R&D SYSTEMS |
| PGP9.5 | - | AB1761-I | MILLIPORE |
| PAR2 | SAM11 | Sc-13504 | Santa Cruz Biotechnology |
| Endothelin 1 | TR.ET.48.5 | MA3-005 | Invitrogen |
| Claudin-1 | - | LS-B6327 | LSBio |
| IL-33 | - | AF3626 | R&D SYSTEMS |

| **Secondary Antibody (Western blot)** | **Clone number** | **Catalog number** | **Brand** |
| --- | --- | --- | --- |
| Mouse IgG | - | PI-2000 | Vector |
| Rabbit IgG | - | PI-1000 | Vector |
| Goat IgG | - | Sc-2768 | Santa Cruz Biotechnology |
| Goat IgG | - | Ab6741 | Abcam |

| **Antibody (Flow cytometry)** | **Clone number** | **Conjugated** | **Brand** |
| --- | --- | --- | --- |
| CD3ε | 124-2C11 | FITC, PE, biotin | BioLegend |
| CD4 | GK1.5 | Biotin | BioLegend |
| CD4 | RM4-5 | PE, Pasific Blue | BioLegend |
| CD4 | RM4-5 | APC | eBioscience |
| CD8 | 53-6.7 | APC, biotin | BioLegend |
| CD11b | M1/70 | APC, PE, biotin | BioLegend |
| CD11c | N418 | FITC, biotin | BioLegend |
| CD11c | HL3 | APC | BD Biosciences |
| CD19 | 6D5 | Biotin | BioLegend |
| CD44 | IM7 | PE-Cy7 | BioLegend |
| CD45 | 30-F11 | PE-Cy7 | BioLegend |
| CD45.2 | 104 | PE-Cy7 | BioLegend |
| CD49b | DX5 | Biotin | BioLegend |
| CD62L | MEL-14 | FITC | BioLegend |
| CD25 | PC61 | APC | BioLegend |
| CD117 | 288 | APC | BioLegend |
| CD127 | A7R34 | PE-Cy7 | BioLegend |
| B220 | RA3-6B2 | FITC, APC | BioLegend |
| FcεR1 | MAR-1 | FITC | BioLegend |
| F4/80 | BM8 | BV421 | BioLegend |
| Gr1 | RB6-8C5 | FITC, biotin | BioLegend |
| NK1.1 | PK136 | PE | BioLegend |
| Sca-1 | E13-161.7 | FITC | BD Biosciences |
| Siglec-F | E50-2440 | PE | BD Biosciences |
| TCRγ/δ | GL3 | BV421, biotin | BioLegend |
| TER119 | TER-119 | Biotin | BD Biosciences |
| GATA3 | TWAJ | PE | eBioscience |

| **Antibody (Naive CD4^+^ T cell sorting assay)** | **Clone number** | **Conjugated** | **Brand** |
| --- | --- | --- | --- |
| CD3 | 145-2C11 | - | BioLegend |
| CD28 | 37.51 | - | BioLegend |
| IL-2 | 200-02 | - | PeproTech |
| IL-4 | 574306 | - | BioLegend |
| IFN-γ | RA6A2 | - | BD Biosciences |
| IL-5 | TRFK5 | PE | BioLegend |
| IL-13 | eBio13A | PE | eBioscience |

**Supplementary Table 3. Primers used for real-time PCR**

| Gene name |  | Primer | |
| --- | --- | --- | --- |
| *Tac1* | Forward | | TCGATGCCAACGATGATCTA |
|  | Reverse | | TCTGCAGAAGATGCTCAAAGG |
| *Ngf* | Forward | | GTGCCTCAAGCCAGTGAAAT |
|  | Reverse | | GTGCCTCAAGCCAGTGAAAT |
| *Klk5* | Forward | | GAAAGTCCTCCAGTGCCTGA |
|  | Reverse | | TAACTTGCCATTGCAGACCA |
| *Klk7* | Forward | | TCTGGCTCCTTTCCCTGATA |
|  | Reverse | | GGTGCGAGCCTTCTTTACAT |
| *Klk14* | Forward | | CTGTTGTCAGATCAATGGGTCATC |
|  | Reverse | | TGAGTCGCTTCCCACCTTCT |
| *Il1β* | Forward | | TGCCACCTTTTGACAGTGATG |
|  | Reverse | | AAGGTCCACGGGAAAGACAC |
| *Il33* | Forward | | CCCGCCTTGCAAAATAAGA |
|  | Reverse | | CTTATGGTGAGGCCAGAACG |
| *St2* | Forward | | CTCCATACAACCACACAATGGA |
|  | Reverse | | CATTTCGACCTTCCTCTTCTTG |
| *Tslp* | Forward | | CGAGCAAATCGAGGACTGTGAG |
|  | Reverse | | GCAGTGGTCATTGAGGGCTTC |
| *Il4* | Forward | | CAAACGTCCTCACAGCAACG |
|  | Reverse | | CATCGAAAAGCCCGAAAGAG |
| *Il13* | Forward | | CTTGCTTGCCTTGGTGGTCT |
|  | Reverse | | ATACCATGCTGCCGTTGCAC |
| *Gapdh* | Forward | | TGTGTCCGTCGTGGATCTGA |
|  | Reverse | | CCTGCTTCACCACCTTCTTGAT |
| *18S rRNA* | Forward | | CGCCCTAGAGGTGAAATTC |
|  | Reverse | | TTGGCAAATGCTTTCGCTC |
