# Supplementary figures and images for: Costello syndrome model mice with a HrasG12S/+ mutation are susceptible to develop house dust mite-induced atopic dermatitis
Source: Cell Death Dis. 2020 Aug 13;11(8):617. doi: 10.1038/s41419-020-02845-8 (PMC7426869; doi:10.1038/s41419-020-02845-8)

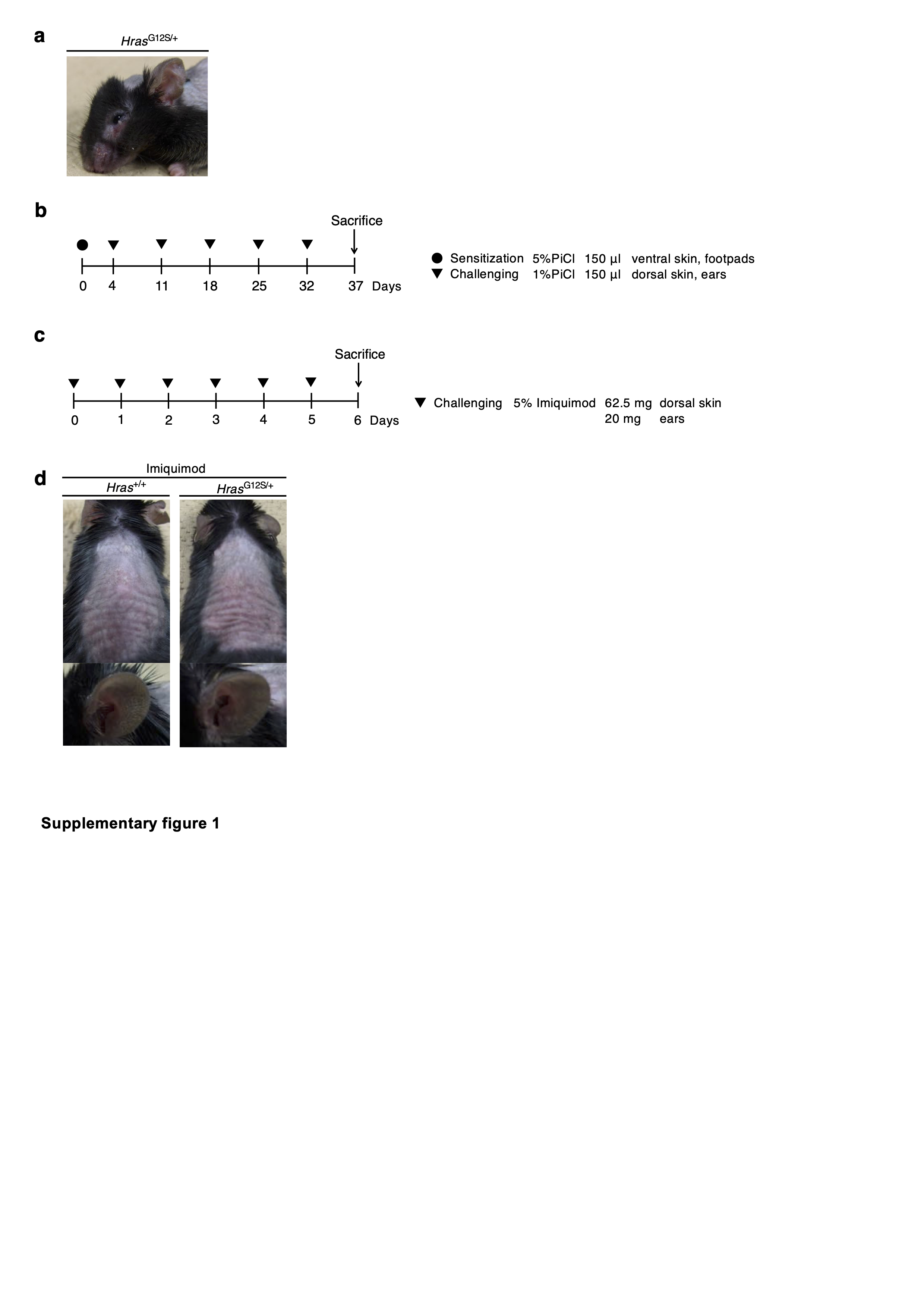

Supplement: Supplementary file 3 — Supplementary Figure 1 [file 41419_2020_2845_MOESM3_ESM.png]

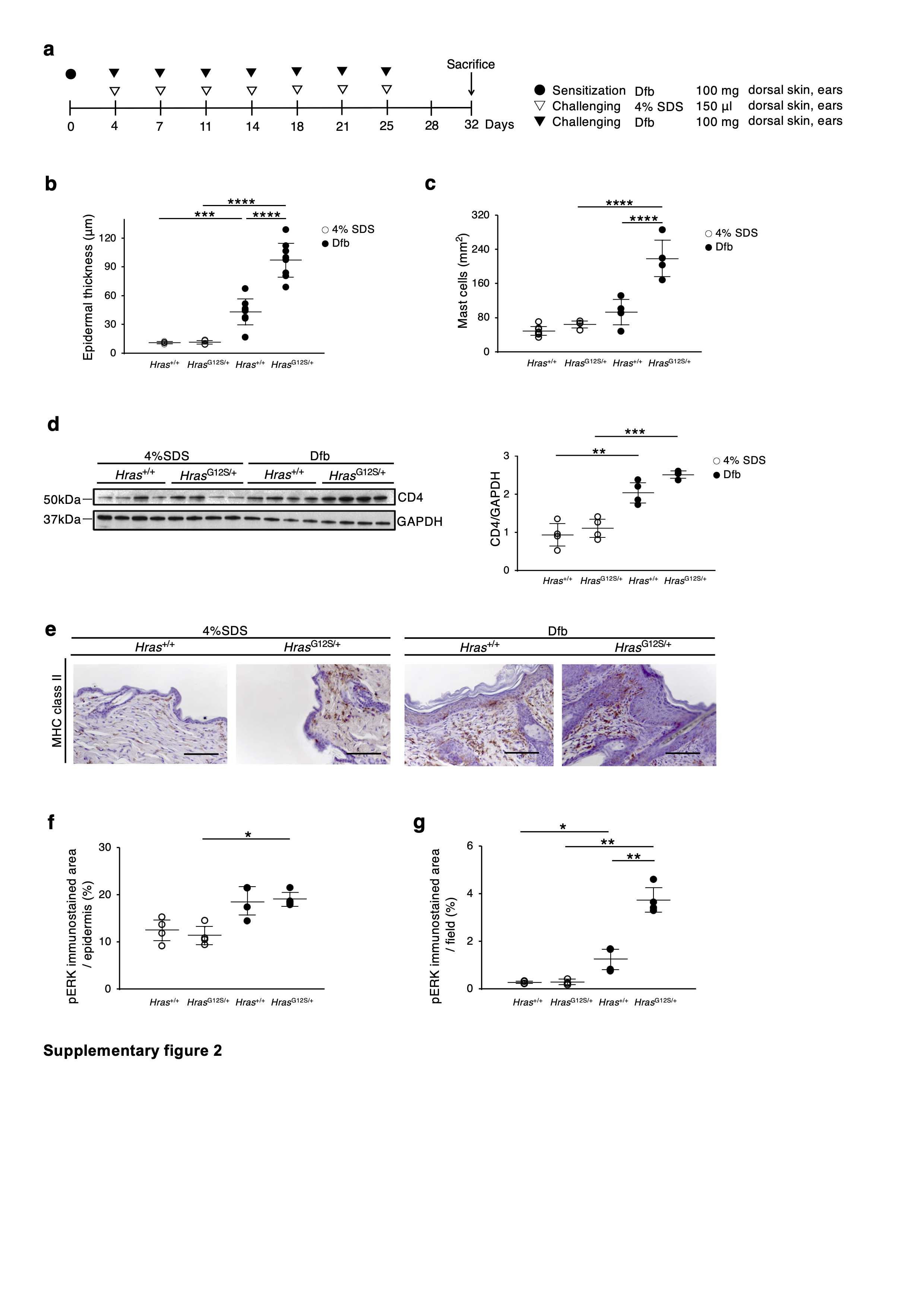

Supplement: Supplementary file 4 — Supplementary Figure 2 [file 41419_2020_2845_MOESM4_ESM.png]

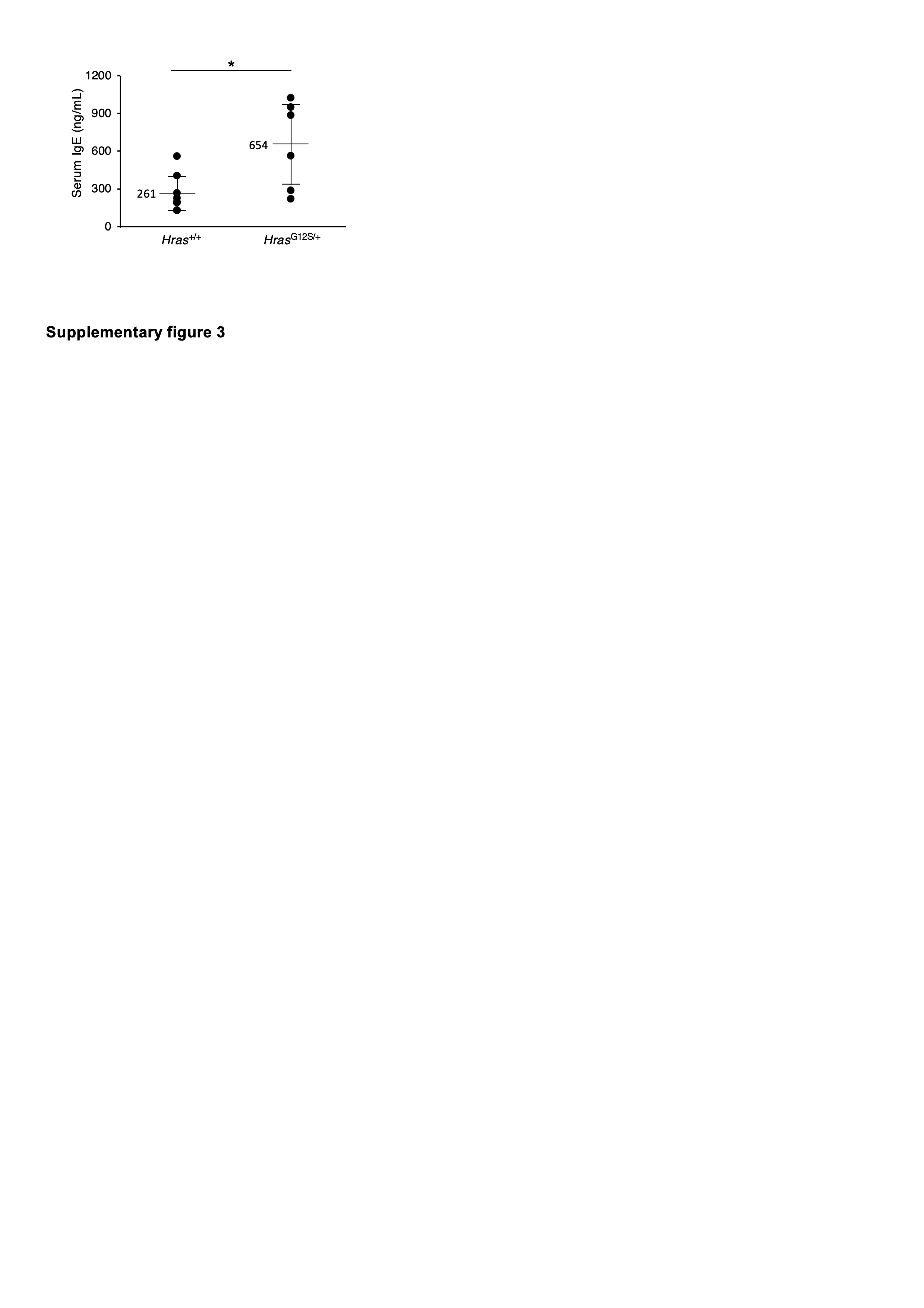

Supplement: Supplementary file 5 — Supplementary Figure 3 [file 41419_2020_2845_MOESM5_ESM.png]

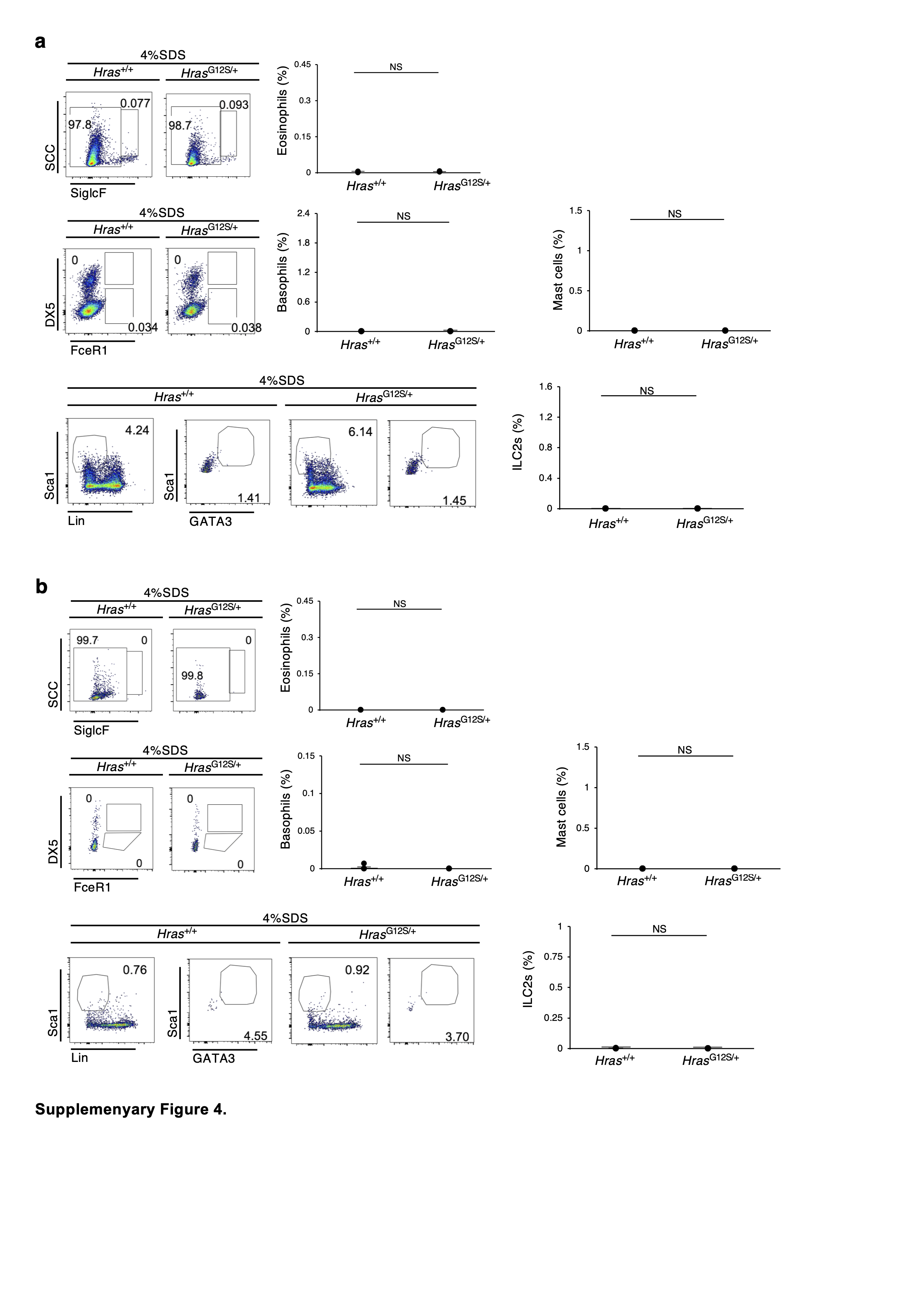

Supplement: Supplementary file 6 — Supplementary Figure 4 [file 41419_2020_2845_MOESM6_ESM.png]

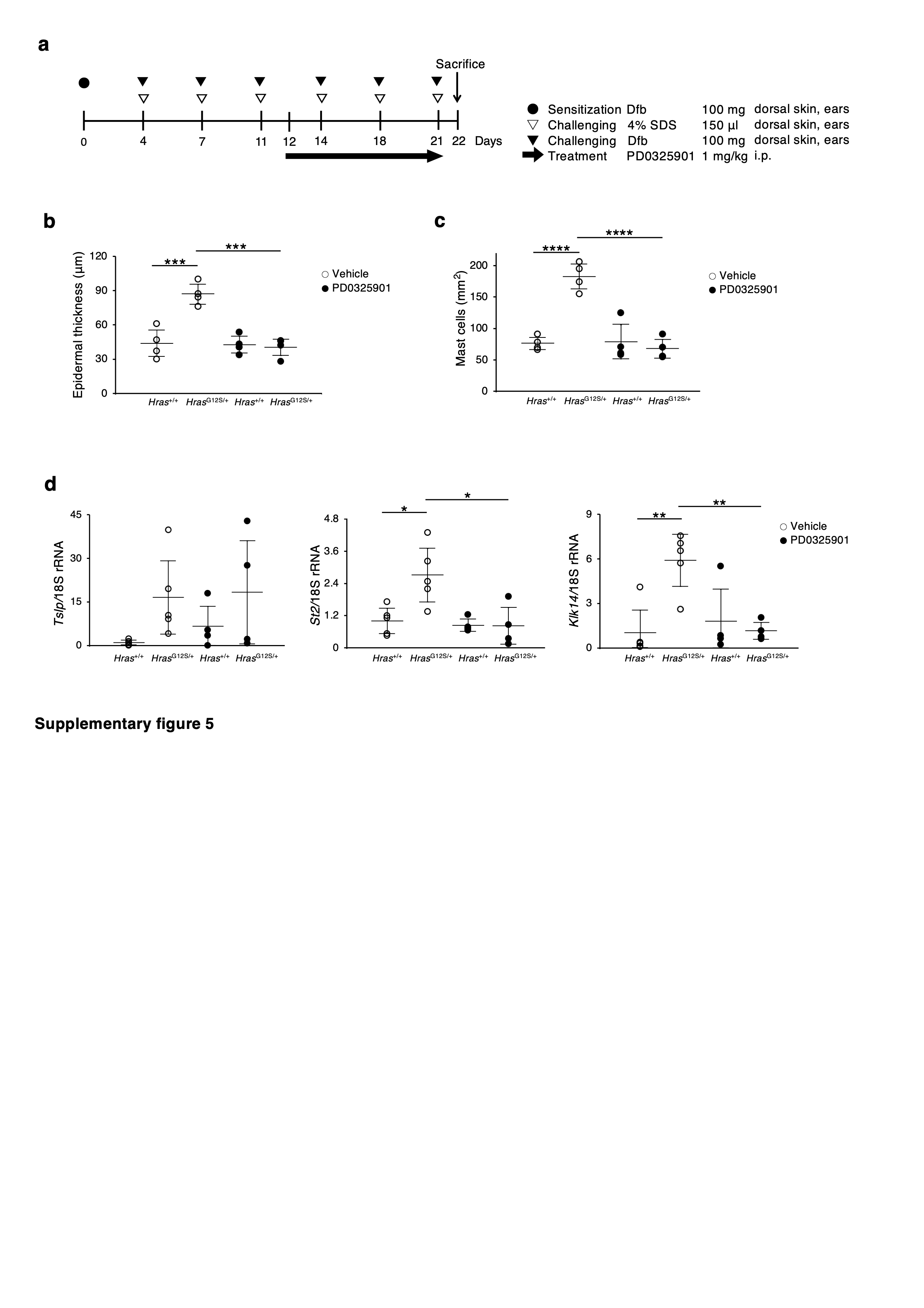

Supplement: Supplementary file 7 — Supplementary Figure 5 [file 41419_2020_2845_MOESM7_ESM.png]
